# Machine Learning Approaches for Predicting Difficult Airway and First-Pass Success in the Emergency Department: Multicenter Prospective Observational Study

**DOI:** 10.2196/28366

**Published:** 2022-01-25

**Authors:** Syunsuke Yamanaka, Tadahiro Goto, Koji Morikawa, Hiroko Watase, Hiroshi Okamoto, Yusuke Hagiwara, Kohei Hasegawa

**Affiliations:** 1 Department of Emergency Medicine & General Internal Medicine The University of Fukui Fukui Japan; 2 Department of Clinical Epidemiology & Health Economics School of Public Health The University of Tokyo Tokyo Japan; 3 Connect Inc Tokyo Japan; 4 Department of Surgery University of Washington Seattle, WA United States; 5 Department of Intensive Care St. Luke's International Hospital Tokyo Japan; 6 Department of Pediatric Emergency and Critical Care Medicine Tokyo Metropolitan Children's Medical Center Tokyo Japan; 7 Department of Emergency Medicine Massachusetts General Hospital Boston, MA United States

**Keywords:** intubation, machine learning, difficult airway, first-pass success

## Abstract

**Background:**

There is still room for improvement in the modified LEMON (look, evaluate, Mallampati, obstruction, neck mobility) criteria for difficult airway prediction and no prediction tool for first-pass success in the emergency department (ED).

**Objective:**

We applied modern machine learning approaches to predict difficult airways and first-pass success.

**Methods:**

In a multicenter prospective study that enrolled consecutive patients who underwent tracheal intubation in 13 EDs, we developed 7 machine learning models (eg, random forest model) using routinely collected data (eg, demographics, initial airway assessment). The outcomes were difficult airway and first-pass success. Model performance was evaluated using c-statistics, calibration slopes, and association measures (eg, sensitivity) in the test set (randomly selected 20% of the data). Their performance was compared with the modified LEMON criteria for difficult airway success and a logistic regression model for first-pass success.

**Results:**

Of 10,741 patients who underwent intubation, 543 patients (5.1%) had a difficult airway, and 7690 patients (71.6%) had first-pass success. In predicting a difficult airway, machine learning models—except for k-point nearest neighbor and multilayer perceptron—had higher discrimination ability than the modified LEMON criteria (all, *P*≤.001). For example, the ensemble method had the highest c-statistic (0.74 vs 0.62 with the modified LEMON criteria; *P*<.001). Machine learning models—except k-point nearest neighbor and random forest models—had higher discrimination ability for first-pass success. In particular, the ensemble model had the highest c-statistic (0.81 vs 0.76 with the reference regression; *P*<.001).

**Conclusions:**

Machine learning models demonstrated greater ability for predicting difficult airway and first-pass success in the ED.

## Introduction

In the emergency department (ED), achieving successful tracheal intubation at the initial attempt (ie, first-pass success) is essential [1]. The literature has shown that repeated intubation attempts are associated with a higher rate of adverse events [2-4]. However, recent studies have also reported first-pass success rates of 74%-84% in the ED [5,6], suggesting that there are still occasions where repeated intubation attempts are required. To improve ED airway management, the development of effective risk stratification and prediction tools is instrumental.

A widely used prediction tool for difficult airway is the modified LEMON (look, evaluate, Mallampati, obstruction, neck mobility) criteria [7], which has been validated [8]. Although the criteria have good prediction ability (eg, sensitivity 86%, specificity 48% for direct laryngoscope) [8], there remains room for improvement. Besides, no prediction tool accurately predicts first-pass success (or failure) in the ED. The recent advent of machine learning approaches has enabled clinicians and researchers to accurately predict various diseases and conditions, such as sepsis [9], acute asthma [10], and ED triage [11,12]. Compared with conventional prediction tools and regression approaches, modern machine learning approaches have several advantages, such as incorporating high-order, nonlinear interactions between predictors and mitigating overfitting [13]. Despite the clinical and research importance, no study has yet applied modern machine learning approaches to predict a difficult airway in advance of preparing for airway management or to predict first-pass success once the intubation strategy has been determined in the ED.

To address this significant knowledge gap in the literature, using data from a prospective, multicenter study of ED airway management, we aimed to develop machine learning models that accurately predict difficult airway and first-pass success and to compare their performance with conventional approaches.

## Methods

### Study Design, Setting, and Participants

This study analyzes data from a multicenter, prospective study of emergency airway management—the second Japanese Emergency Airway Network (JEAN-2) study. The details of the study design, setting, participants, methods of data measurement, and definitions of variables have been reported elsewhere [14]. In brief, the JEAN-2 study is a consortium of 13 academic and community EDs, including 10 level I and 3 level Ⅱ equivalent trauma centers. These EDs are located in different geographic regions across Japan. The median ED census is 29,000 patients per year (range of 16,000 to 67,000 annual visits). These ED are affiliated with an emergency medicine residency training program. Attending physicians or resident physicians who are under the supervision of the attending physician perform intubations. In this observational study, patients were managed at the discretion of treating physicians. The institutional review board at each participating center approved the waiver of informed consent before data collection. This study used data from consecutive (both children and adults) patients who underwent ED management at one of the participating EDs from January 1, 2010 through December 31, 2018. Patients who underwent surgical intubations at the first attempt were excluded.

### Outcomes

The outcomes of interests were difficult airway and first-pass success. According to the American Society of Anesthesiologists (ASA) guidelines, a difficult airway was defined as multiple intubation attempts by emergency physicians or anesthesiologists according to the ASA guidelines [15]. First-pass success was defined as intubation success at the initial attempt of each encounter [16]. Intubation success was defined as the proper placement of a tracheal tube through the vocal cord, confirmed by the use of quotative or end-tidal CO_2_ monitoring [17]. An intubation attempt was defined as a single insertion of the laryngoscope past the teeth [18].

### Predictors of Machine Learning Models

To develop machine learning models for the difficult airway outcome, we used the following variables that are *routinely* obtained in advance of the actual intubation attempt: patient demographics (age, sex, estimated height and body weight, BMI), components of the modified LEMON criteria, pre-intubation vital signs (pulse rate, systolic blood pressure, respiratory rate, oxygen saturation), and Glasgow coma scale. To develop models that predict the first-pass success outcome (once the intubation strategy has been determined), we used all available intubation-related information—in addition to the aforementioned predictors—such as type of day (weekend/weekday), medications, intubation methods, intubation devices, intubator’s post-graduate year, and intubator’s specialty.

### Statistical Analysis

Summary statistics were used to describe the characteristics of patients and airway management. After performing imputations for missing continuous variables (most predictors had <10% missingness; Multimedia Appendix 1) using random forest [19], we conducted predictor preprocessing, such as one-hot encoding (ie, creation of dummy variables), normalization, and standardization. The nonlinear predictors included in the developed models were age, body weight, height, BMI, and pre-intubation vital signs. In the training set (80% random sample), for each outcome, we developed 7 machine learning models: (1) logistic regression model with elastic-net (penalized logistic regression) [20], (2) random forest [21], (3) gradient boosting decision tree [22], (4) multilayer perceptron [23], (5) k-point nearest neighbor [24], (6) XGBoost [25], and (7) ensemble model (ridge regression and the random forest with an equal weight) [26]. For the difficult airway outcome, the modified LEMON criteria model was used as the reference model. For the first-pass success outcome, a (nonpenalized) logistic regression model was used as the reference model. We performed stratified 5-fold cross-validation to determine the optimal hyperparameters with the highest c-statistic (ie, the area under the receiver operating characteristic [ROC] curve).

In the test set (the remaining 20% of the random sample), we measured the performance of reference and machine learning models. We estimated the c-statistic of each model and examined the following association measures: sensitivity, specificity, positive and negative predictive values, and positive and negative likelihood ratios. The c-statistic is the probability that, given 2 individuals (one who experiences the outcome of interest and the other who does not), the model estimates a higher probability for the first patient than for the second [27]. We determined the threshold of perspective prediction (cut-off) results based on the ROC curve from the Youden method [28]. For the model with the highest c-statistic among the 7 machine learning models, we computed the variable importance—how strongly each of the predictors improved the c-statistic. We also examined calibration plots of the best-performing machine learning model for each of the outcomes. Data were analyzed using python (version 3.7.3) and R (version 3.6.2). Two-sided *P* values <.05 were considered statistically significant.

## Results

### Patient Characteristics

During the 108-month study period, the JEAN-2 study recorded data for 10,816 patients (capture rate, 96%) who underwent emergency airway management at one of the 13 participating EDs. Of these, 75 patients who underwent surgical intubation at their first attempt were excluded; the remaining 10,741 patients comprised the analytic cohort. The patient characteristics, details of airway management, and intubation outcomes are shown in Table 1. The median age was 71 (IQR 56 -81) years, 2.8% (304/10,741) were children, and 38.0% (4079/10,741) were female. Overall, 5.1% (543/10,741) of patients had a difficult airway outcome, while 71.6% (7690/10,741) had first-pass success. An aborted intubation attempt occurred for 39 patients.

**Table 1:** Patient characteristics, airway management, and outcomes in 10,741 patients who underwent tracheal intubation in the emergency department.

Variables			Results
Age (years), median (IQR)			71 (56-81)
Children ( 18 years), n (%)			304 (2.8)
Female gender, n (%)			4079 (38.0)
Estimated height (cm), median (IQR)			160 (153-170)
Estimated body weight (kg), median (IQR)			60 (50-67)
BMI (kg/m^2^), median (IQR)			22.0 (19.5-24.3)
**Primary indication, n (%)**			
	Medical cardiac arrest	3785 (35.2)	
	Traumatic cardiac arrest	438 (4.1)	
	Medical indication	5440 (50.6)	
	Airway problem (eg, obstruction)	289 (2.7)	
	Breathing problem (eg, respiratory failure)	1673 (15.6)	
	Circulation problem (eg, shock)	1080 (10.1)	
	Altered mental status	2036 (19.0)	
	Others	360 (3.4)	
	Traumatic indication	1080 (10.1)	
**Modified LEMON^a^ criteria, n (%)**			
	Look externally	583 (5.0)	
	3-3-(2) rule	3620 (33.7)	
	Obstruction	774 (7.2)	
	Neck mobility	1101 (10.3)	
	Any criterion met in the modified LEMON criteria	4709 (43.8)	
**Intubation outcomes, n (%)**			
	Difficult airway	543 (5.1)	
	First-pass success	7690 (71.6)	

^a^LEMON: look, evaluate, Mallampati, obstruction, neck mobility.

### Prediction Performance for Difficult Airway Outcomes

**Figure 1:**
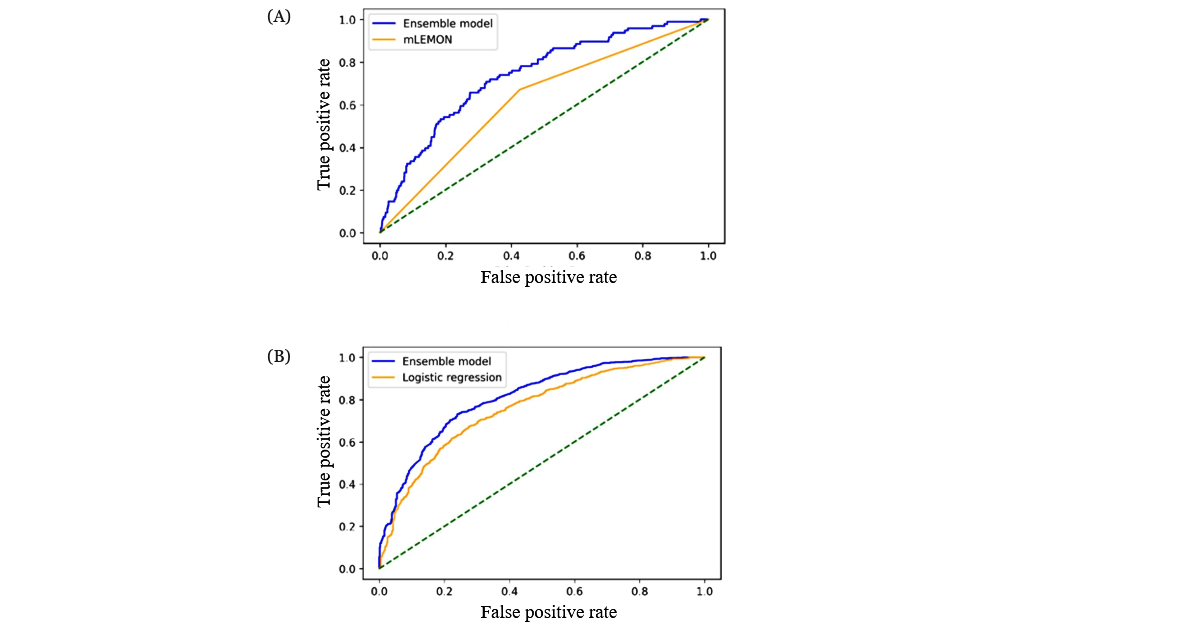
Discrimination ability of the ensemble model and logistic regression (reference) model in predicting intubation outcomes, including (A) difficult airway outcomes and (B) first-pass success outcomes. mLEMON: modified look, evaluate, Mallampati, obstruction, neck mobility model.

**Figure 2:**
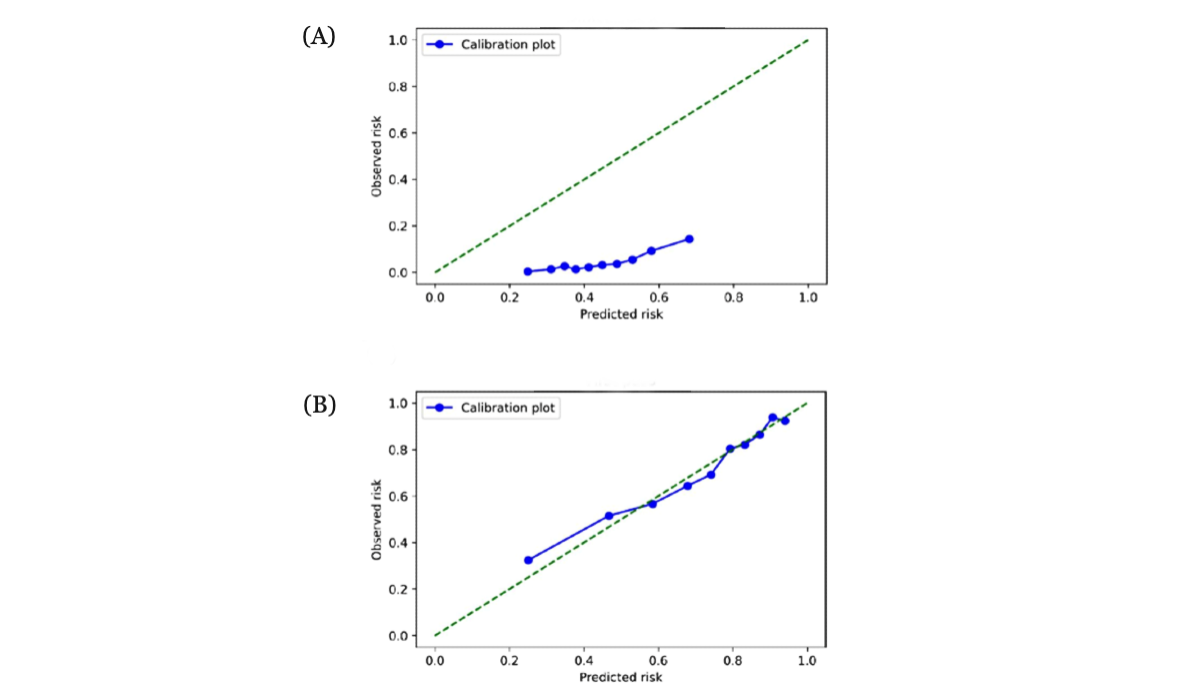
Calibration plots of ensemble models in predicting intubation outcomes, including (A) difficult airway outcomes and (B) first-pass success outcomes.

Table 2 summarizes the performance of the modified LEMON criteria (reference) and 7 machine learning models when predicting a difficult airway outcome in the ED. Compared with the modified LEMON criteria, the discrimination ability of machine learning models—except for the k-point nearest-neighbor model and multilayer perceptron model—were significantly greater (*P*≤.001). Among the 7 machine learning models, the ensemble model had the highest c-statistic (0.74, 95% CI 0.67-0.79; Figure 1A), with a sensitivity of 0.67 (95% CI 0.65-0.69), specificity of 0.70 (95% CI 0.68-0.72), positive predictive value of 0.09 (95% CI 0.08-0.11), and negative predictive value of 0.98 (95% CI 0.97-0.98). Compared with the modified LEMON criteria, which had a specificity of 0.57 (95% CI 0.56-0.58), all machine learning models had higher specificity, with the multilayer perceptron model achieving a specificity of 0.92 (95% CI 0.90-0.93). The calibration plot (Figure 2A)—which indicates how far the predicted risk from the ensemble model deviated from the actual risk—showed that the ensemble model overestimated the risk of the outcome, while there was a positive relationship between the predicted and actual risks, largely due to the class imbalance (ie, difficult airway outcome occurred only in 5% of the sample).

**Table 2:** Performance of 7 machine learning models and modified LEMON (look, evaluate, Mallampati, obstruction, neck mobility) criteria when predicting difficult airway outcome in the emergency department.

Models	C-statistic^a^ (95% CI)	*P* value	Sensitivity (95% CI)	Specificity (95% CI)	PPV^b^ (95% CI)	NPV^c^ (95% CI)	PLR^d^ (95% CI)	NLR^e^ (95% CI)
Modified LEMON criteria (reference)	0.62 (0.60-0.64)	Reference^f^	0.67 (0.66-0.68)	0.57 (0.56-0.58)	0.08 (0.07-0.08)	0.97 (0.97-0.97)	1.57 (1.48-1.68)	0.57 (0.54-0.61)
Penalized logistic regression	0.73 (0.68-0.79)	<.001	0.66 (0.64-0.68)	0.68 (0.66-0.70)	0.09 (0.08-0.10)	0.98 (0.97-0.98)	2.05 (1.75-2.40)	0.51 (0.43-0.59)
Random forest	0.72 (0.67-0.77)	<.001	0.58 (0.56-0.60)	0.74 (0.72-0.75)	0.09 (0.08-0.11)	0.97 (0.97-0.98)	3.84 (2.50-5.90)	0.84 (0.55-1.29)
Gradient boost	0.72 (0.66-0.77)	.001	0.77 (0.75-0.79)	0.58 (0.56-0.60)	0.08 (0.07-0.09)	0.98 (0.98-0.99)	1.84 (1.63-2.08)	0.39 (0.35-0.44)
Multilayer perceptron	0.57 (0.50-0.63)	.14	0.19 (0.17-0.20)	0.92 (0.90-0.93)	0.09 (0.08-0.11)	0.96 (0.95-0.97)	2.24 (1.44-3.48)	0.89 (0.57-1.38)
K-point nearest neighbor	0.54 (0.49-0.61)	.02	0.39 (0.36-0.41)	0.70 (0.68-0.72)	0.06 (0.05-0.07)	0.96 (0.95-0.97)	1.30 (1.00-1.68)	0.87 (0.67-1.14)
XGBoost	0.72 (0.67-0.77)	<.001	0.69 (0.67-0.71)	0.60 (0.58-0.62)	0.07 (0.06-0.09)	0.98 (0.97-0.98)	1.70 (1.47-1.97)	0.52 (0.45-0.61)
Ensemble model^g^	0.74 (0.67-0.79)	<.001	0.67 (0.65-0.69)	0.70 (0.68-0.72)	0.09 (0.08-0.11)	0.98 (0.97-0.98)	2.21 (1.89-2.58)	0.48 (0.41-0.56)

^a^C-statistic in the modified LEMON was evaluated using 95% CIs.

^b^PPV: positive predictive value.

^c^NPV: negative predictive value.

^d^PLR: positive likelihood ratio.

^e^NLR: negative likelihood ratio.

^f^Comparison of the area under the curve of the reference model (modified LEMON) with that of each machine learning model using the DeLong test.

^g^Ensemble prediction model using these machine learning models (that combined ≥2 models).

### Prediction Performance for First-Pass Success Outcomes

Table 3 summarizes the performance of the reference model and 7 machine learning models when predicting the first-pass success outcome in the ED. Compared with the reference model, the discrimination ability of machine learning models—except for the random forest and k-point nearest neighbor models—was significantly higher (all *P*<.05). Among the 7 machine learning models, the ensemble model had the highest c-statistic (0.81, 95% CI 0.79-0.83; Figure 1B). Compared with the reference model, the ensemble model had a higher sensitivity (0.79, 95% CI 0.77-0.81) and specificity (0.67, 95% CI 0.65-0.69), with a PPV of 0.85 (95% CI 0.84-0.87) and NPV of 0.57 (95% CI 0.55-0.59). Compared with the reference model, which had a specificity of 0.36 (95% CI 0.34-0.38), most machine learning models had higher specificity, with the random forest model achieving a specificity of 0.70 (95% CI 0.68-0.72). In the calibration plot of the ensemble model (Figure 2B), the model-predicted probability was well-matched with the observed probabilities.

**Table 3:** Performance of 7 machine learning models and reference model when predicting first-pass success outcome in the emergency department.

Models	C statistic (95% CI)	*P* value	Sensitivity (95% CI)	Specificity (95% CI)	PPV^a^ (95% CI)	NPV^b^ (95% CI)	PLR^c^ (95% CI)	NLR^d^ (95% CI)
Logistic regression (reference)	0.76 (0.74-0.78)	(Reference)^e^	0.91 (0.89-0.92)	0.36 (0.34-0.38)	0.78 (0.76-0.79)	0.61 (0.59-0.63)	1.42 (1.33-1.51)	0.26 (0.24-0.27)
Penalized logistic regression	0.81 (0.79-0.83)	.001	0.79 (0.77-0.80)	0.70 (0.68-0.72)	0.86 (0.85-0.88)	0.57 (0.55-0.59)	2.59 (2.29-2.92)	0.31 (0.27-0.35)
Random forest	0.78 (0.76-0.81)	.12	0.78 (0.76-0.79)	0.64 (0.62-0.66)	0.84 (0.83-0.86)	0.54 (0.52-0.56)	2.16 (1.94-1.2.41)	0.35 (0.31-0.39)
Gradient boost	0.80 (0.78-0.82)	.005	0.92 (0.91-0.94)	0.40 (0.38-0.43)	0.79 (0.77-0.81)	0.69 (0.67-0.71)	1.55 (1.45-1.66)	0.19 (0.17-0.20)
Multilayer perceptron	0.81 (0.79-0.83)	.002	0.92 (0.91-0.93)	0.44 (0.42-0.46)	0.80 (0.78-0.82)	0.69 (0.67-0.71)	1.64 (1.53-1.76)	0.18 (0.17-0.19)
K-point nearest neighbor	0.75 (0.73-0.77)	.60	0.98 (0.97-0.98)	0.18 (0.16-0.20)	0.74 (0.73-0.76)	0.78 (0.76-0.80)	1.19 (1.15-1.24)	0.12 (0.11-0.12)
XGBoost	0.81 (0.79-0.83)	<.001	0.94 (0.93-0.95)	0.38 (0.36-0.40)	0.79 (0.77-0.81)	0.73 (0.71-0.75)	1.53 (1.43-1.62)	0.15 (0.14-0.16)
Ensemble model^f^	0.81 (0.79-0.83)	<.001	0.79 (0.77-0.81)	0.67 (0.65-0.69)	0.85 (0.84-0.87)	0.57 (0.55-0.59)	2.39 (2.13-2.67)	0.31 (0.28-0.35)

^a^PPV: positive predictive value.

^b^NPV: negative predictive value.

^c^PLR: positive likelihood ratio.

^d^NLR: negative likelihood ratio.

^e^Comparison of the area under the curve of the reference model with that of each machine learning model using the DeLong test.

^f^Ensemble prediction model using these machine learning models (that combined ≥2 models).

### Variable Importance

Table 4 shows the variable importance of the best performance model (the ensemble model) for each outcome. For the difficult airway prediction, the most contributing predictor was age, followed by any criterion met in the modified LEMON criteria and hyoid mental distance ≥3 fingers. For the first-pass success prediction, the best contributing predictor was the use of laryngeal pressure, followed by lifting force required for laryngeal deployment and Cormack grade of 3.

**Table 4:** Importance of each predictor of ensemble model when predicting difficult airway and first-pass success outcomes.

Predictors			Δ c-statistics^a^
**Predictors for difficult airway outcome**			
	Age	0.093	
	Any modified LEMON^b^ criterion met	0.093	
	Hyoid mental distance ≥3 fingers	0.091	
	Interincisor distance of 3 fingers	0.084	
	BMI	0.080	
	Body weight	0.073	
	>80 years old	0.070	
	Hyoid mental distance of 2 fingers	0.053	
	Airway obstruction	0.049	
	Neck mobility	0.048	
**Predictors for first-pass success outcome**			
	Use of laryngeal pressure	0.118	
	Lifting force required for laryngeal deployment	0.108	
	Cormack grade of 3	0.099	
	Any modified LEMON criterion met	0.094	
	Cormack grade of 1	0.094	
	Intubator’s post-graduation year of 1 or 2	0.090	
	Neuromuscular blocking agent (rocuronium)	0.077	
	Rapid sequence intubation	0.076	
	Video laryngoscope (C-MAC)	0.075	
	Video Cormack grade of 1	0.074	
	Interincisor distance ≥3 fingers	0.079	

^a^The variable importance of a predictor is agnostic regarding the direction of the association.

^b^LEMON: look, evaluate, Mallampati, obstruction, neck mobility.

## Discussion

### Principal Findings

In this analysis of multicenter prospective data from 10,741 ED patients, we applied modern machine learning models to predict intubation-related outcomes in the ED. Specifically, compared with conventional approaches (ie, modified LEMON criteria and nonpenalized logistic regression model), most machine learning models demonstrated superior discrimination performance when predicting both difficult airway and first-pass success outcomes. Additionally, these machine learning models also achieved higher specificity when predicting these 2 outcomes. To the best of our knowledge, this is the first study that has investigated the performance of modern machine learning models when predicting clinically important intubation outcomes in the ED setting.

Consistent with our findings, the following has been reported as predictors for first-pass success in the ED: patient characteristics (eg, restricted mouth opening, restricted neck extension, and swollen tongue), high Cormack grade, intubators’ characteristics (eg, clinical experience and working department), the use of rapid-sequence intubation, and the use of video laryngoscope at the first attempt [6,29-31].

The importance of accurate prediction for difficult airways has been emphasized in ED airway management [8]. Although the modified LEMON criteria (and the LEMON criteria) have been validated as an indicator for difficult airways, their prediction ability is suboptimal for clinical use [7,8]. In the operating room setting, a couple of studies have reported a potential benefit of machine learning models for predicting difficult airways [32,33]. For example, in a single-center study of 80 patients, a deep learning approach using data from the patients’ facial images had high discrimination ability for difficult airways—defined as multiple attempts by an intubator with at least 12 months of anesthesia experience, grade 3 or 4 laryngoscopic view, need for a second intubator, or nonelective use of an alternative airway device [32]. Our multicenter study—with a sample size that is many times larger than the prior studies on this topic—builds on these earlier reports and extends them by demonstrating that modern machine learning models outperform conventional approaches for predicting intubation outcomes in the ED.

The observed improvement in prediction ability by machine learning approaches may be explained by several reasons. First, the machine learning approaches account for high-order interactions between predictors and nonlinear relationships with an outcome, which traditional modeling approaches cannot address [34]. Second, the modified LEMON criteria may be too parsimonious (ie, the use of a limited number of predictors), while the applied machine learning models could use a larger number of predictors. Third, the modern machine learning approaches enabled us to minimize overfitting, such as lasso and ridge penalizations (ie, elastic net model and cross-validation). In addition to these strengths, modern machine learning models also are scalable for further improvement by integration with recently developed techniques such as image analysis of patients’ faces and necks [32,35].

Although the machine learning models achieved a more significant predictive ability, their performance remained imperfect. This may be explained, at least partially, by the limited set of predictors (eg, lack of detailed information on the intubation competency and experience of each intubator) and data measurement errors. Additionally, one may surmise that the modified LEMON criteria are simpler and easier to use in the ED. Despite the known trade-off between parsimonious models and more complex models with a larger number of predictors, the use of modern machine learning models has advantages in the era of health information technology, including automated data entry through voice recognition, natural language processing, continuous sophistication of models through sequential extractions of electronic health records, and reinforcement learning [36,37]. Our findings and the recent advent of machine learning approaches collectively support cautious optimism that machine learning may enhance the clinician’s ability—as assistive technology—to predict patient outcomes in the ED. The resulting accurate prediction of intubation outcomes has several important implications in airway practice in the ED. For example, early identification of difficult airways should help ED providers develop individualized and optimal management strategies and prepare for rescue airways [14,38]. Besides, the accurate estimation of the probability of first-pass success given the conditions (eg, the airway management strategies and intubator to be used) would not only increase the opportunity for clinical training (eg, which patient can be safely intubated by the intubator) but also improve patient safety.

To implement our developed machine learning models, a web-based application or integrated emergency department information system is needed. The rapid development of health information technology (eg, web-based artificial intelligence application with the model) enables us to implement the developed model into the real clinical setting. Furthermore, the current models can be used not only for practice but as an educational tool. For example, in simulation-based intubation training, supervisors can evaluate the trainee’s intubation strategy by indicating the actual probability of difficult airway and first-pass success.

### Limitations

Several potential limitations of this study should be noted. First, our data may be subject to self-reporting and measurement bias (eg, underreporting difficult airways). However, the study was conducted by investigators using a standardized protocol [6], which led to the high capture rate (96%) and low proportion of missingness in the predictors and outcomes (Multimedia Appendix 1). Second, we did not have detailed information on the procedural competency of each intubator, as this factor is also challenging to define and measure in real-world settings. To address this issue, we used years of experience and specialty, which are readily available in most ED settings, as a proxy for the competency. Third, machine learning models have a common limitation in the interpretability of models. Fourth, because of the small samples of children (2.8%), our model may not have optimal prediction ability in pediatric populations. Finally, our models may not be generalizable to other practice settings, although the study sample consisted of a geographically diverse patient across Japan.

### Conclusions

In summary, based on the extensive multicenter, prospective data from 10,741 ED intubations, we developed modern machine learning models to predict clinically essential intubation outcomes. Using routinely available data as the predictors, we found that the machine learning models had a greater ability to predict difficult airways and first-pass success than conventional approaches. Although formal validation is required, this study lends support to the application of machine learning models for the prediction of intubation-related outcomes, which will, in turn, improve airway management practice and outcomes of critically ill patients in the ED.

## Appendix

Multimedia Appendix 1 Proportion of missingness in predictors used in machine learning models.

## Abbreviations

ASA: American Society of Anesthesiologists
ED: emergency department
JEAN-2: second Japanese Emergency Airway Network
ROC: receiver operating characteristic

## References

[1] Goto T, Goto Y, Hagiwara Y, Okamoto H, Watase H, Hasegawa K (2019). Advancing emergency airway management practice and research. Acute Med Surg.

[2] Hasegawa K, Shigemitsu K, Hagiwara Y, Chiba T, Watase H, Brown CA, Brown DFM, Japanese Emergency Medicine Research Alliance Investigators (2012). Association between repeated intubation attempts and adverse events in emergency departments: an analysis of a multicenter prospective observational study. Ann Emerg Med.

[3] Walls RM, Brown CA, Bair AE, Pallin DJ, NEAR II Investigators (2011). Emergency airway management: a multi-center report of 8937 emergency department intubations. J Emerg Med.

[4] Pallin DJ, Dwyer RC, Walls RM, Brown CA, NEAR III Investigators (2016). Techniques and trends, success rates, and adverse events in emergency department pediatric intubations: a report from the National Emergency Airway Registry. Ann Emerg Med.

[5] Park L, Zeng I, Brainard A (2017). Systematic review and meta-analysis of first-pass success rates in emergency department intubation: Creating a benchmark for emergency airway care. Emerg Med Australas.

[6] Goto Y, Goto T, Hagiwara Y, Tsugawa Y, Watase H, Okamoto H, Hasegawa K, Japanese Emergency Medicine Network Investigators (2017). Techniques and outcomes of emergency airway management in Japan: An analysis of two multicentre prospective observational studies, 2010-2016. Resuscitation.

[7] Reed MJ, Dunn MJG, McKeown DW (2005). Can an airway assessment score predict difficulty at intubation in the emergency department?. Emerg Med J.

[8] Hagiwara Y, Watase H, Okamoto H, Goto T, Hasegawa K, Japanese Emergency Medicine Network Investigators (2015). Prospective validation of the modified LEMON criteria to predict difficult intubation in the ED. Am J Emerg Med.

[9] Islam MM, Nasrin T, Walther BA, Wu C, Yang H, Li Y (2019). Prediction of sepsis patients using machine learning approach: A meta-analysis. Comput Methods Programs Biomed.

[10] Goto T, Camargo CA, Faridi MK, Yun BJ, Hasegawa K (2018). Machine learning approaches for predicting disposition of asthma and COPD exacerbations in the ED. Am J Emerg Med.

[11] Goto T, Camargo CA, Faridi MK, Freishtat RJ, Hasegawa K (2019). Machine learning-based prediction of clinical outcomes for children during emergency department triage. JAMA Netw Open.

[12] Raita Y, Goto T, Faridi MK, Brown DFM, Camargo CA, Hasegawa K (2019). Emergency department triage prediction of clinical outcomes using machine learning models. Crit Care.

[13] Kotsiantis S, Zaharakis I, Pintelas P, Maglogiannis I, Karpouzis K, Wallace BA, Soldatos J (2007). Supervised machine learning: A review of classification techniques. Emerging Artificial Intelligence Applications in Computer Engineering: Real Word AI Systems with Applications in eHealth, HCI, Information Retrieval and Pervasive Technologies.

[14] Goto T, Gibo K, Hagiwara Y, Morita H, Brown DF, Brown CA, Hasegawa K, Japanese Emergency Medicine Network Investigators (2015). Multiple failed intubation attempts are associated with decreased success rates on the first rescue intubation in the emergency department: a retrospective analysis of multicentre observational data. Scand J Trauma Resusc Emerg Med.

[15] Apfelbaum J, Hagberg C, Caplan R, Blitt C, Connis R, Nickinovich D, Hagberg CA, Caplan RA, Benumof JL, Berry FA, Blitt CD, Bode RH, Cheney FW, Connis RT, Guidry OF, Nickinovich DG, Ovassapian A, American Society of Anesthesiologists Task Force on Management of the Difficult Airway (2013). Practice guidelines for management of the difficult airway: an updated report by the American Society of Anesthesiologists Task Force on Management of the Difficult Airway. Anesthesiology.

[16] Goto T, Gibo K, Hagiwara Y, Okubo M, Brown DFM, Brown CA, Hasegawa K (2016). Factors associated with first-pass success in pediatric intubation in the emergency department. West J Emerg Med.

[17] Link MS, Berkow LC, Kudenchuk PJ, Halperin HR, Hess EP, Moitra VK, Neumar RW, O’Neil BJ, Paxton JH, Silvers SM, White RD, Yannopoulos D, Donnino MW (2015). Part 7: adult advanced cardiovascular life support. Circulation.

[18] Hasegawa K, Hagiwara Y, Chiba T, Watase H, Walls RM, Brown DF, Brown CA, Japanese Emergency Medicine Research Alliance (2012). Emergency airway management in Japan: Interim analysis of a multi-center prospective observational study. Resuscitation.

[19] Stekhoven D (2012). Nonparametric missing value imputation using random forest.

[20] Warton DI (2012). Penalized normal likelihood and ridge regularization of correlation and covariance matrices. Journal of the American Statistical Association.

[21] Svetnik V, Liaw A, Tong C, Culberson JC, Sheridan RP, Feuston BP (2003). Random forest: a classification and regression tool for compound classification and QSAR modeling. J Chem Inf Comput Sci.

[22] Natekin A, Knoll A (2013). Gradient boosting machines, a tutorial. Front Neurorobot.

[23] Pal S, Mitra S (1992). Multilayer perceptron, fuzzy sets, and classification. IEEE Trans Neural Netw.

[24] Bay S (1999). Nearest neighbor classification from multiple feature subsets. Intelligent Data Analysis.

[25] Chen T, He T, Benesty M, Khotilovich V, Tang Y (2015). Xgboost: extreme gradient boosting. R package version.

[26] Ruta D, Gabrys B (2005). Classifier selection for majority voting. Information Fusion.

[27] Pencina MJ, D'Agostino RB (2015). Evaluating discrimination of risk prediction models: the c statistic. JAMA.

[28] Taner T, Antony J (2000). The assessment of quality in medical diagnostic tests: a comparison of ROC/Youden and Taguchi methods. Int J Health Care Qual Assur Inc Leadersh Health Serv.

[29] Sagarin MJ, Barton ED, Chng Y, Walls RM, National Emergency Airway Registry Investigators (2005). Airway management by US and Canadian emergency medicine residents: a multicenter analysis of more than 6,000 endotracheal intubation attempts. Ann Emerg Med.

[30] Jung W, Kim J (2020). Factors associated with first-pass success of emergency endotracheal intubation. Am J Emerg Med.

[31] Taboada M, Soto-Jove R, Mirón P, Martínez S, Rey R, Ferreiroa E, Almeida X, Álvarez J, Baluja A (2019). Evaluation of the laryngoscopy view using the modified Cormack-Lehane scale during tracheal intubation in an intensive care unit. A prospective observational study. Rev Esp Anestesiol Reanim (Engl Ed).

[32] Connor C, Segal S (2011). Accurate classification of difficult intubation by computerized facial analysis. Anesth Analg.

[33] Moustafa MA, El-Metainy S, Mahar K, Mahmoud Abdel-magied E (2019). Defining difficult laryngoscopy findings by using multiple parameters: A machine learning approach. Egyptian Journal of Anaesthesia.

[34] Kuhn M, Johnson K (2013). Applied predictive modeling.

[35] An G, Omodaka K, Tsuda S, Shiga Y, Takada N, Kikawa T, Nakazawa T, Yokota H, Akiba M (2018). Comparison of machine-learning classification models for glaucoma management. J Healthc Eng.

[36] Freeman MB (2001). Method and apparatus for automated data entry. Justia Patents.

[37] Thanaki J (2017). Python natural language processing.

[38] Lockey D, Crewdson K, Weaver A, Davies G (2014). Observational study of the success rates of intubation and failed intubation airway rescue techniques in 7256 attempted intubations of trauma patients by pre-hospital physicians. Br J Anaesth.

