# Supplementary material for: Machine Learning Approaches for Predicting Difficult Airway and First-Pass Success in the Emergency Department: Multicenter Prospective Observational Study
Source: Interact J Med Res. 2022 Jan 25;11(1):e28366. doi: 10.2196/28366 (PMC8826144; doi:10.2196/28366)
Supplement: Multimedia Appendix 1 [file ijmr_v11i1e28366_app1.docx]

**Supplemental Table 1. Proportion of missingness in predictors used in machine learning models**

| **Predictors** | **Missing data (%)** |
| --- | --- |
| **Patients characteristics** |  |
| Age | 26 (0.2) |
| Sex | 0 (0) |
| Estimated height | 631 (5.8) |
| Estimated body weight | 594 (5.5) |
| Body mass index | 702 (6.4) |
| Primary indication | 0 (0) |
| Modified LEMON criteria |  |
| Look externally | 336 (3.2) |
| Inter-incisor distance | 2470 (23.0) |
| Hyoid mental distance | 2391 (22.2) |
| Obstruction | 379 (3.5) |
| Neck mobility | 507 (4.7) |
| Vital signs before intubation attempt |  |
| Pre-intubation heart rate | 172 (1.6) |
| Pre-intubation blood pressure | 348 (3.2) |
| Pre-intubation respiratory rate | 729 (6.7) |
| Pre-intubation saturation | 648 (6.0) |
| Pre-intubation Glasgow Coma Scale | 226 (2.1) |
| **Airway management characteristics** |  |
| Intubation date | 0 (0) |
| Intubation method | 3 (0) |
| First intubation device | 17 (0.2) |
| Cormack grade with a direct laryngoscope | 3377 (31.4) |
| Cormack grade with a video laryngoscope | 1060 (9.9) |
| Lifting force required for laryngeal deployment | 354 (3.3) |
| Use of laryngeal pressure | 430 (4.0) |
| Vocal code mobility | 532 (5.0) |
| First intubator’s specialty | 33 (0.3) |
| First intubator’s post-graduation year | 58 (0.5) |

Abbreviation: LEMON, look-evaluate-Mallampati-obstruction-neck mobility
